# Excessive recreational computer use and food consumption behaviour among adolescents

**DOI:** 10.1186/1824-7288-36-52

**Published:** 2010-08-05

**Authors:** Lu Shi, Yuping Mao

**Affiliations:** 1 Department of Health Services, 650 Charles E. Young Drive S. 61-253 CHS, University of California Los Angeles, Los Angeles, CA 90095, USA; 2 Department of Communications and Technology, Enterprise Square, University of Alberta, 10230 Jasper Avenue, Edmonton, Alberta, T5J 4P6, Canada

## Abstract

**Introduction:**

Using the 2005 California Health Interview Survey (CHIS) data, we explore the association between excessive recreational computer use and specific food consumption behavior among California's adolescents aged 12-17.

**Method:**

The adolescent component of CHIS 2005 measured the respondents' average number of hours spent on viewing TV on a weekday, the average number of hours spent on viewing TV on a weekend day, the average number of hours spent on playing with a computer on a weekday, and the average number of hours spent on playing with computers on a weekend day. We recode these four continuous variables into four variables of "excessive media use," and define more than three hours of using a medium per day as "excessive." These four variables are then used in logistic regressions to predict different food consumption behaviors on the previous day: having fast food, eating sugary food more than once, drinking sugary drinks more than once, and eating more than five servings of fruits and vegetables. We use the following variables as covariates in the logistic regressions: age, gender, race/ethnicity, parental education, household poverty status, whether born in the U.S., and whether living with two parents.

**Results:**

Having fast food on the previous day is associated with excessive weekday TV viewing (O.R. = 1.38, p < 0.01). Having sugary food more than once is associated with excessive weekend TV viewing (O.R. = 1.50, p < 0.001). Having sugary drinks more than once is associated with excessive weekday TV viewing (O.R. = 1.41, p < 0.01), excessive weekday recreational computer use (O.R. = 1.38, p < 0.05), and excessive weekend TV viewing (O.R. = 1.43, p < 0.001). Finally, having more than five servings of fruits and vegetables on the previous day is negatively associated with all four media use variables: excessive weekday TV viewing (O.R. = 0.64, p < 0.001), excessive weekday recreational computer use (O.R. = 0.68, p < 0.01), excessive weekend TV viewing (O.R. = 0.80, p < 0.05), and excessive weekend recreational computer use (O.R. = 0.78, p < 0.05).

**Conclusion:**

Excessive recreational computer use independently predicts undesirable eating behaviors that could lead to overweight and obesity. Preventive measures ranging from parental/youth counseling to content regulations might be addressing the potential undesirable influence from excessive computer use on eating behaviors among children and adolescents.

## Introduction

A possible association between one's excessive computer use and his or her obesity risk has been noted in earlier studies [1,2]. However, the exact pathways between computer use and obesity had remained unclear. While one's time spent in front of the computer screen might be at the expense of physical activity (the "physical activity displacement hypothesis"), it is also very likely that Internet food advertisements lure people to make suboptimal food choices and in turn increased their bodyweight [3] (the "media change intake" hypothesis). Meanwhile, eating and drinking in front of a screen might easily promote snacking and overeating [4-6]. A necessary condition for the "media change intake" hypothesis to hold is the evidence for an association between specific food intake patterns and time spent on computer use, yet no study to date has looked at this type of associational patterns. To fill this research gap, this paper explores whether excessive computer predicts consumption of fast food, sugar-sweetened beverage, high-sugar foods, and fruits and vegetables.

## Method

We use the 2005 adolescent sample of California Health Interview Survey (CHIS), the only population survey dataset we know to have measured both media consumption behavior and food consumption patterns among adolescents, to explore the association between excessive recreational computer use and specific food consumption. CHIS is a biennial population health survey [7], and its adolescent sample is collected via telephone interviews with adolescents living in sampled households.

The adolescent component of CHIS 2005 measured the respondents' average number of hours spent on viewing TV on a weekday, the average the average number of hours spent on viewing TV on a weekend day, the average number of hours spent on playing with a computer on a weekday, and the average number of hours spent on playing with computers on a weekend day. We recode these four continuous variables into four dummy variables of "excessive media use," and define more than three hours of using a medium per day as "excessive" since this is the threshold where media consumption predicts health-related behavior [8]. These four dummy variables are then used in logistic regressions to predict different food consumption behaviors on the previous day. While the key independent variables here are excessive weekday recreational computer use and excessive weekend recreational computer use, the two variables about excessive TV viewing are used as covariates to control for their confounding effects. We use four logistic regressions to predict the following four food consumption behaviors:

1. Having had fast food the day before (recoded from the CHIS survey item "Yesterday, how many times did you eat fast food? Include fast food meals eaten at school, at home or at fast-food restaurants, carryout or drive thru.")

2. Having had sugary food more than once the day before (recoded from the survey item "How many servings of high sugar foods, such as cookies, candy, doughnuts, pastries, cake or popsicles did you have?")

3. Having had sugary drinks more than once the day before (recoded from the survey item "Yesterday, how many glasses or cans of soda, such as Coke, or other sweetened drinks, such as fruit punch or Sunny Delight did you drink? Do not count diet drinks.")

4. Having had five or more servings of fruits and vegetables as per the Center of Disease Control and Prevention instruction [9] (recoded from two survey items: "Yesterday, how many servings of fruit, such as an apple or banana did you eat?" and "Yesterday, how many servings of vegetables, like corn, green beans, green salad, or other vegetables did you eat?").

We use the following variables as covariates in the four logistic regressions: age, gender, race/ethnicity, parental education, household poverty status (below federal poverty line = 1), whether born in the United States [10], and whether living with two parents [11].

## Results

Table 1 lists the descriptive statistics for the predictor variables. Among the 4029 adolescents surveyed, 39.0% reported having fast food on the previous day, 33.8% reported having high-sugar food on the previous day, 30.3% reported having sugary drink on the previous day, and 25.0% reported having five servings of fruits and vegetables on the previous day.

**Table 1:** Demographic and Behavioural Profile of the Study Sample

Variable	Percent/mean (standard error)
Male	50.9%
	
Age	14.41(.026)
	
Race/ethnicity	
White	53.3%
Latino	21.1%
Asian	8.8%
Black	5.8%
Other	11.0%
	
Parental education	
Less than high school	36.9%
High school	21.3%
Some college	26.4%
College graduate	15.5%
	
Below federal poverty line	12.0%
	
Living with two parents	69.8%
	
Born in the United States	90.2%
	
Food/beverage consumption the day before	
Had fast food	39.0%
Had high-sugar food more than once	33.8%
Had sugary drinks more than once	30.6%
Had fruits and vegetable for five servings or more	25.0%
	
Daily hrs on TV-video/weekday	1.97 (.026)
	
Daily hrs on computer/weekday	1.36 (.025)
	
Daily hrs on TV-video/weekend	2.77 (.035)
	
Daily hrs on computer/weekend	1.61 (.030)

*N *= 4029

Table 2 shows the logistic regression results for the four models. Having fast food on the previous day is associated with excessive weekday TV viewing (O.R. = 1.38, p < 0.01), which means for an adolescent who viewed more than three hours of TV per weekday the odds of having fast food is 38% bigger than those who did not view as much. Having sugary food more than once is associated with excessive weekend TV viewing (O.R. = 1.50, p < 0.001). Having sugary drinks more than once is associated with excessive weekday TV viewing (O.R. = 1.41, p < 0.01), excessive weekday recreational computer use (O.R. = 1.38, p < 0.05), and excessive weekend TV viewing (O.R. = 1.43, p < 0.001). Finally, having more than five servings of fruits and vegetables on the previous day is negatively associated with all four media use variables: excessive weekday TV viewing (O.R. = 0.64, p < 0.001), excessive weekday recreational computer use (O.R. = 0.68, p < 0.01), excessive weekend TV viewing (O.R. = 0.80, p < 0.05), and excessive weekend recreational computer use (O.R. = 0.78, p < 0.05).

**Table 2:** Logistic Regressions of Food Consumption among Adolescents in California (N = 4029)

	Eating fast food yesterday	High-sugar food more than once yesterday	Sugary drink more than once yesterday	> 5 servings of fruit/vegetable yesterday
	Odds Ratios	Odds Ratios	Odds Ratios	Odds Ratios
Gender (female = 1)	0.91	1.15*	0.61**	0.98
Age	1.05**	0.98	1.09**	0.89**
Parental Education				
*Less than high school*	Ref.	Ref.	Ref.	Ref.
*High school*	1.17	0.95	1.20	0.85
*Some college*	1.17	0.89	0.98	0.76**
*College graduate*	0.73*	1.02	0.63**	1.25*
Race/Ethnicity				
*White*	Ref.	Ref.	Ref.	Ref.
*Latino*	1.54**	0.91	1.37**	0.90
*Black*	1.46**	1.22	1.63**	0.64**
*Asian*	1.18	0.84	0.77	1.36**
*American Indian*	1.13	1.20	1.31*	1.03
*Pacific Islander*	1.45	0.83	0.73	0.70
*Other*	1.34*	1.05	1.53**	0.77*
Below poverty line	0.87	1.05	0.77*	1.07
Born in the US	1.02	1.06	1.29	0.93
Living with two parents	1.01	1.10	0.86	1.19*
TV > 3 hrs weekday	1.38**	1.20	1.41**	0.64**
Computer > 3 hrs weekday	1.11	1.08	1.38*	0.68**
TV > 3 hrs weekend day	1.09	1.50**	1.43**	0.80**
Computer > 3 hrs weekend day	1.20	1.02	1.21	0.78*

Note:

*: p-value is less than .05

**: p-value is less than .01

## Discussion

As our logistic regressions show, excessive recreational computer use independently predicts undesirable eating behaviors that could lead to overweight and obesity [12,13], even when we control for television viewing and socio-demographic covariates. These results are consistent with previous studies of television viewing and unhealthy eating behavior [6,14,15]. The literature on television and unhealthy intake indicated that sugary food and beverage are more likely to reach the young audience and change their intake behavior via advertisement than fruits and vegetables [16-18]. This mechanism of "media content changes behavior" might also explain the association between recreational computer use and unhealthy intake as well, as Internet food advertisements show a similar content pattern to that of television commercials [3]. Another explanation for the association between sugary drinks and excessive computer use on weekdays could that the long duration of gaming in front of a screen necessitates the intake of caffeinated drinks like coca-cola [19], especially if the computer use occurs during weekdays when adolescents are more likely to play video games or web games at night. In other words, drinking caffeinated sugary drinks might enable gamers to stay longer in front of the computer screen and hence the association between sugary drink consumption and the time spent on recreational computer use.

This is the first study, to the best of our knowledge, to explore the association between recreational computer use and specific food/drink consumption patterns among adolescents. As the society attempts to address the digital divide by providing more computer access to younger populations, the content of computer games and the Internet remains largely unregulated as compared with traditional media like TV. The traditional media channels like TV now have various restrictions and technical devices to limit children's exposure to food commercials, yet much less attention has been paid to the food advertisement on the Internet, a media channel extremely popular among younger populations. Moreover, if children who are at risk of being overweight are more likely to live in households where parents might have less technical knowledge and time available to monitor children's computer use, then the introduction of computers and the Internet could have magnified the existing disparities in the current obesity epidemic. Thus, it might be advisable to adopt more preventive measures, including youth counseling and content regulations, to address the potential undesirable influence from excessive computer use on eating and drinking behaviors among children and adolescents.

## Competing interests

The authors declare that they have no competing interests.

## Authors' contributions

Lu Shi contributed the data analysis and Yuping Mao contributed the literature review. All authors have read and approved the final manuscript.
